# *Aspergillus niger* Spores Are Highly Resistant to Space Radiation

**DOI:** 10.3389/fmicb.2020.00560

**Published:** 2020-04-03

**Authors:** Marta Cortesão, Aram de Haas, Rebecca Unterbusch, Akira Fujimori, Tabea Schütze, Vera Meyer, Ralf Moeller

**Affiliations:** ^1^Space Microbiology Research Group, Radiation Biology Department, Institute of Aerospace Medicine, German Aerospace Center, Cologne, Germany; ^2^Department of Basic Medical Sciences for Radiation Damages, National Institutes for Quantum and Radiological Science and Technology, Chiba, Japan; ^3^Chair of Applied and Molecular Microbiology, Institute of Biotechnology, Technische Universität Berlin, Berlin, Germany

**Keywords:** *Aspergillus niger*, *A. niger* spores, spore survival, space, radiation, X-ray, UV, international space station

## Abstract

The filamentous fungus *Aspergillus niger* is one of the main contaminants of the International Space Station (ISS). It forms highly pigmented, airborne spores that have thick cell walls and low metabolic activity, enabling them to withstand harsh conditions and colonize spacecraft surfaces. Whether *A. nige*r spores are resistant to space radiation, and to what extent, is not yet known. In this study, spore suspensions of a wild-type and three mutant strains (with defects in pigmentation, DNA repair, and polar growth control) were exposed to X-rays, cosmic radiation (helium- and iron-ions) and UV-C (254 nm). To assess the level of resistance and survival limits of fungal spores in a long-term interplanetary mission scenario, we tested radiation doses up to 1000 Gy and 4000 J/m^2^. For comparison, a 360-day round-trip to Mars yields a dose of 0.66 ± 0.12 Gy. Overall, wild-type spores of *A. niger* were able to withstand high doses of X-ray (LD_90_ = 360 Gy) and cosmic radiation (helium-ion LD_90_ = 500 Gy; and iron-ion LD_90_ = 100 Gy). Drying the spores before irradiation made them more susceptible toward X-ray radiation. Notably, *A. niger* spores are highly resistant to UV-C radiation (LD_90_ = 1038 J/m^2^), which is significantly higher than that of other radiation-resistant microorganisms (e.g., *Deinococcus radiodurans*). In all strains, UV-C treated spores (1000 J/m^2^) were shown to have decreased biofilm formation (81% reduction in wild-type spores). This study suggests that *A. niger* spores might not be easily inactivated by exposure to space radiation alone and that current planetary protection guidelines should be revisited, considering the high resistance of fungal spores.

## Introduction

Radiation is the most challenging factor for life in the space environment (Horneck et al., 2010; Chancellor et al., 2014). On the one hand, the Sun emits UV radiation (non-ionizing), X-rays (ionizing electromagnetic waves) and solar flares (intense bursts of high-energy ionizing radiation) (Sliney, 2007). On the other hand, cosmic events such as supernova explosions or pulsars, emit galactic cosmic radiation (GCR) (Chancellor et al., 2018). GCR particle spectrum spans from light particles, such as hydrogen-ions (85%) and helium-ions (He, 14%), to high charge Z and energy particles (HZE) like iron-ions (Fe, 0.03%) (Horneck et al., 2010). Radiation shielding on the International Space Station (ISS) is provided by both the Earth’s magnetosphere and the walls of the space station. However, not all types of radiation are easily shielded. For instance, HZE particles are still capable of penetrating current space vehicles (Chancellor et al., 2018). Protecting living systems from radiation becomes particularly challenging beyond low Earth orbit (LEO). Due to the absence of Earth’s magnetosphere, space missions toward the Moon or Mars will be exposed to substantially higher radiation doses than those currently experienced on the ISS (Cucinotta et al., 2013; Chancellor et al., 2014, 2018; Narici et al., 2017). Studies on how radiation affects cells have identified two main types of damage: direct and indirect. Direct damage targets DNA (e.g., single- or double-strand breaks), proteins, or lipids. Whereas indirect damage is induced by the generation of reactive oxygen species (ROS) – which are produced by the interaction of radiation with cellular water molecules in a process called radiolysis (Cadet et al., 2015; Moeller et al., 2017).

Regardless of the damage, many microorganisms, especially spore formers, are able to withstand high radiation doses (Horneck et al., 2010; Moeller et al., 2010). Spores of the bacterium *Bacillus subtilis* are known to be highly resistant to extreme space conditions and therefore are currently being used as indicators for decontamination protocols and planetary protection policies (Kminek et al., 2019). However, while survival of bacterial spores has been extensively studied in both Earth and spaceflight contexts (Moeller et al., 2014; Setlow, 2014; Khodadad et al., 2017), survival of fungal spores has not. Samples from the ISS indoor microbiome identified *Aspergillus niger* as one of the most common fungal contaminants (Novikova et al., 2006; Checinska et al., 2015). Contrary to *B. subtilis* spores, which are formed as a response to stressful conditions, asexual spores of *A. niger* (i.e., conidia) are produced as a natural part of its life cycle (Krijgsheld et al., 2013). *A. niger* spores are highly pigmented and can be easily dispersed through the air which facilitates habitat colonization. Also, as an opportunistic human pathogen, inhalation of *A. niger* spores can lead to human respiratory infections (Silverman et al., 1967; Latgé, 1999; Esbelin et al., 2013). Thus, the ability of *A. niger* to survive and grow in the spaceflight environment is a potential threat to both astronaut health and spacecraft safety. Nonetheless, *A. niger* is also a well-established cell factory used in modern-day biotechnology to produce various compounds such as proteins, enzymes, and pharmaceuticals (Meyer et al., 2015; Cairns et al., 2018). This makes *A. niger* a potential asset in long-term space missions, where astronauts will have to produce their own compounds of interest such as vitamins or antibiotics (Cortesão et al., 2020).

Despite the relevance of *A. niger* spores in the space context, it is not yet known whether they are able to withstand extreme space radiation conditions. Fungal spore survival generally depends on two main components. One is the spore cell wall, which helps to prevent radiation damage on the DNA. The spore cell wall is composed of polysaccharides (mainly chitin and glucans), and is covered by an outer layer of rodlets (hydrophobins) and pigments (e.g., melanin). These outer layers of the cell wall make spores highly hydrophobic and highly pigmented (Beauvais et al., 2014). Pigments, such as melanin, are known to be involved in different cellular processes, from adhesion to virulence, as well as to protect cells from radiation-induced stress and ROS (Cockell and Knowland, 1999; Eisenman and Casadevall, 2012; Cordero and Casadevall, 2017). Melanized fungi have been reported in Chernobyl sites (Zhdanova et al., 2000; Casadevall et al., 2017), and some were even found displaying increased growth after X-ray irradiation (Dadachova et al., 2007). Previous studies have reported the presence of melanin in *A. niger* spores as an adaptive trait conferring resistance toward UV-A (315–400) (Singaravelan et al., 2008). Moreover, studies on clinical isolates of *Aspergillus fumigatus* reported the involvement of DHN-melanin in UV-C protection. Here, loss of a polyketide synthase from the DHN-melanin pathway (Δ*pksP*) resulted in decreased survival, when exposed to 100 J/m^2^. This was not the case for an *A. fumigatus* strain isolated from the ISS, where loss of *pksP* did neither reduce nor increase viability (Blachowicz et al., 2020). However, a recent review emphasizes that pigment biosynthesis in *Aspergillus* species is not yet fully understood (Chang et al., 2020). Pigmentation in *A. niger* is particularly puzzling. The pigment spectrum of *A. niger* spores was shown to have two main absorbance peaks, which together absorb light in the entire VIS-spectrum and thus result in the black color. These are thought to be two distinct components – one green (peak at ∼575 nm), and one brown component (∼425 nm) – and were both shown to be FwnA dependent (Jorgensen et al., 2011). FwnA is an ortholog of pksP, and deletion of the *fwnA* gene (Δ*fwnA*) results in fawn-colored (not white as for *A. fumigatus*) spores. Knowing if and how pigmentation is involved in spore resistance will be crucial to understand the limits of spore survival, which will, in turn, help develop adequate decontamination approaches. Another important component in fungal spore resistance is DNA repair. When damage occurs, several pathways can be activated: nucleotide excision repair (NER), mismatch repair (MMR), homologous recombination (HR), or non-homologous end-joining (NHEJ) recombination (Sinha and Hader, 2002). *A. niger* strains deficient in NHEJ (Δ*kusA*) are widely used to generate mutant strains, but it was also shown that these strains are more sensitive to UV and X-ray irradiation (Meyer et al., 2007).

When considering fungal contamination in indoor habitats, the ability to colonize is not only dependent on spore survival, but also on the ability for spores to adhere to a surface, germinate, and grow. Germination and hyphal growth are established through polarized growth (Kwon et al., 2011, 2013). In *A. niger*, the stabilization of polarity axes during germination is dependent on the Rho GTPase RacA. A *racA* deletion displays a hyperbranching phenotype which results in compact colonies (Kwon et al., 2011). Spore adhesion to surfaces is facilitated by proteins in the cell wall that help fungi to grow on a wide-range of substrates (e.g., from quartz used on windows to silicone and polycarbonate used in medical/scientific instruments) (Makimura et al., 2001; Mora et al., 2016, 2019). Furthermore, fungal growth is surface-associated, which can induce biocorrosion. In fact, fungal-induced biocorrosion has led to major problems in spacecraft safety such as those in the Mir space station (Klintworth and Reher, 1999; Novikova et al., 2006). Understanding whether polar growth impacts spore revival and subsequent surface-associated growth is important to better control fungal contamination in the spaceflight context.

For these reasons, understanding whether *A. niger* spores resist to space radiation, and to what extent, will be crucial to assess both the risks and opportunities of fungal spore survival during space travel. This study has assessed *A. niger* spore survival to different types of space radiation (X-rays, cosmic radiation, and UV-C). Three mutant strains were included to elucidate the role of pigmentation (Δ*fwnA*) and DNA repair (Δ*kusA*) on spore resistance to radiation. In addition, high radiation doses were tested to assess the limits of resistance of fungal spores and their survival potential during long-term space travel. In addition, a fourth strain deficient in polar growth (Δ*racA*) was tested to assess the impact of UV-C treatment in spore revival and consequent ability for surface colonization.

## Materials and Methods

### Strains and Media

Aspergillus niger wild-type (N402) and three mutant strains with defects in pigmentation (ΔfnwA), DNA repair (ΔkusA), and polar growth control (ΔracA), were used in this study and are listed in Table 1. A. niger spores were harvested from 3-day-old cultures incubated on complete medium (CM) at 30°C [55 mM glucose, 11 mM KH_2_PO_4_, 7 mM KCl, 178 nM H_3_BO_3_, 2 mM MgSO_4_, 76 nM ZnSO_4_, 70 mM NaNO_3_, 6.2 nM Na_2_MoO_4_, 18 nM FeSO_4_, 7.1 nM CoCl_2_, 6.4 nM CuSO_4_, 25 nM MnCl_2_, 174 nM EDTA, 0.5% (w/v) yeast extract and 0.1% (w/v) casamino acids] by flooding the agar plates with saline solution (0.9% NaCl) and harvesting the spores using a cotton stick. Spore suspensions were filtered using Miracloth (Millipore) to remove hyphal fragments and were kept at 4°C. Counting was done using a Neubauer chamber. All experiments were performed with spore suspensions not older than 2 weeks. Viability assays were done using minimal medium (MM) [55 mM glucose, 11 mM KH_2_PO_4_, 7 mM KCl, 178 nM H_3_BO_3_, 2 mM MgSO_4_, 76 nM ZnSO_4_, 70 mM NaNO_3_, 6.2 nM Na_2_MoO_4_, 18 nM FeSO_4_, 7.1 nM CoCl_2_, 6.4 nM CuSO_4_, 25 nM MnCl_2_, 174 nM EDTA]. For MM or CM agar plates, 15 g agar was added per liter (adapted from Carvalho et al., 2010).

**TABLE 1:** *Aspergillus niger* strains used in the study.

Name	Strain	Relevant genotype	Description	References
Wild-type	N402		Wild-type strain capable of DNA repair and pigment formation which give black-colored spores	Bos et al., 1988
Color mutant	MA93.1	Δ*fwnA*	Loss of pigment, due to lack of polyketide synthase results in fawn-colored spores	Jorgensen et al., 2011
NHEJ mutant	MA78.6	Δ*kusA*	Inactive in NHEJ pathway and thus impaired in DNA repair	Meyer et al., 2007
Polar growth mutant	MA80.1	Δ*kusA*, Δ*racA*	Inactive in NHEJ pathway and polar growth control	Kwon et al., 2011

### X-Ray Radiation Exposure

Spores of *A. niger* strains were exposed to X-ray radiation in PCR tubes (Brand), each filled with 100 μl of saline solution (0.9% NaCl) at a concentration of 10^7^ spores/ml. This concentration was chosen after testing the effect of initial spore concentration (inoculum) on survivability toward X-rays (Supplementary Figure 1). Radiation exposure was performed using the RS225 X-ray machine (Gulmay Medical Systems, Camberley, Surrey, United Kingdom) operated without filter at 200 kV and 15 mA which allowed exposure of high doses in a short amount of time. Dose rate, in Gy/min, was determined using the UNIDOS webline with an ionization chamber type TM30013 (PTW, Freiburg, Germany). For each desired dose, the sample exposure time was adjusted given that distance and dose rate were kept constant. X-ray exposure time was calculated as follows:

$$t\,\left(\text{min}\right)=\frac{R\,\left(\text{Gy}\right)}{d\,\left(\text{Gy}/\text{min}\right)}$$

where *t* = time (in minutes); *R* = desired radiation dose (in Gy), *d* (dosimeter value in Gy/min). Samples were exposed to 50, 100, 250, 500, and 1000 Gy. Given that the average dose rate was ∼20 Gy/min, the maximum time a sample was exposed to X-ray radiation was 50 min (corresponding to 1000 Gy). To test irradiation of dried spores, 25 μl of a spore suspension (to a total of 10^7^ spores per PCR tube) either in water (H_2_O) or in saline solution (0.9% NaCl) was placed in PCR tubes, which were left to air-dry overnight on the bench (22°C) before irradiation. After irradiation, the spores were suspended in 100 μl of water (H_2_O) or saline solution (0.9% NaCl). Radiation exposure included at least three biological replicates per strain and was performed two independent times (*n* = 6). Viability was determined by colony forming units (CFUs) (see section “Viability Assay”).

### Cosmic Radiation Exposure

Spores were exposed to helium- and iron-ions (two components of cosmic radiation) in PCR tubes (Brand) each filled with 100 μl of saline solution (0.9% NaCl) at a concentration of 10^7^ spores/ml. PCR tubes were placed inside Petri dishes stacked together inside plastic bags and placed directly facing the ion beam. Samples were exposed to 10, 100, 250, and 500 Gy. Non-irradiated controls were left at room temperature. Viability was calculated by CFU (see section “Viability Assay”). Radiation exposure included three biological replicates per strain (*n* = 3). Cosmic radiation exposure was performed at the Heavy Ion Medical Accelerator (HIMAC) facility at the National Institute of Radiological Sciences (NIRS) in Chiba, Japan. The helium-ion beam had an energy of 150 MeV/n and linear energy transfer (LET) of 2.2 keV/μm. The iron-ion beam had an energy of 500 MeV/n and LET of 200 keV/μm. Each tested dose was adjusted by exposure time, since beam energy and LET was kept constant.

### UV-C Radiation Exposure

Spores of A. niger were exposed to UV radiation in Petri dishes with an initial concentration of 10^6^ spores/ml in 15 ml of saline solution (0.9% NaCl). The concentration of 10^6^ spores/ml ensures a spore monolayer and prevents survival due to shielding by the spores themselves. The UV lamp (MagneTel, Menomonee Falls, WI, United States) was used a UV-C monochromatic wavelength of 254 nm. During irradiation, magnetic stirrers continuously mixed the spore suspension in order to avoid mutual shielding of the spores. The radiation dose [J/m^2^] was adjusted through exposure time, since the height of the UV lamp was kept constant. For that, UV fluence was determined using the dosimeter (UVP UVX radiometer), and exposure time was adjusted for each sample to reach each desired UV dose (i.e., 150, 250, 500, 1000, 2000, 3000, and 4000 J/m^2^). UV-C exposure time was calculated as follows:

$$t\,\left(\text{s}\right)=\frac{R\,\left(J/m^{2}\right)\times 100}{d\,\left(\mu \,\text{W}/\text{c}\,{\text{m}}^{2}\right)}$$

where *t* = time (in seconds); *R* = desired radiation dose (in J/m^2^), *d* (dosimeter value for UV fluence, in μW/cm^2^). After each time point, corresponding to a certain dose, 100 μl of sample were taken in triplicate from the spore suspension and transferred into PCR tubes. Viability was calculated by CFU (see section “Viability Assay”). Radiation exposure included at least three biological replicates per strain and was performed two independent times (*n* = 6).

### Oxidative Stress Assay

Oxidative stress resistance toward hydrogen peroxide (H_2_O_2_) was measured using a protocol adapted from Riesenman and Nicholson (2000). Spores of *A. niger* were diluted to a final concentration of 10^8^ spores/ml in saline solution (0.9% NaCl), 833 μl of which were placed in a 5 ml tube (Eppendorf). Afterward 167 μl of 30% H_2_O_2_ were added (Sigma-Aldrich). The spore-H_2_O_2_ suspension was gently mixed at RT (∼22°C) and incubated for up to 15 min in a final concentration of 5% H_2_O_2_. 30 μl of the suspension were taken at different time points and diluted 1:10 in saline solution (0.9% NaCl) with bovine catalase (100 μg/ml) to stop the oxidation reaction. Samples were serially diluted and used to determine viability (see section “Viability Assay”). This assay included three biological replicates per tested strain, and was performed two independent times (*n* = 6).

### Viability Assay

Viability of *A. niger* spores was determined by their ability to form colonies after exposure to the tested environments. Samples were serially diluted up to 10^–8^ using a 96-well plate, each well with a total volume of 300 μl. To count the CFUs, 20 μl of each dilution was plated out in triplicate on 1/8 of a Petri dish with MM agar, containing Triton X-100 (0.05%) to facilitate counting. The plates were incubated for 2 days at 30°C before the colonies were counted. This allowed calculation of the survival fraction ratio (N/N0, in which N is the number of CFU of the treated samples and N0 that of the controls). To analyze the microscopic morphology, previously irradiated samples were diluted 100-fold before plating 10 μl on a MM agar plate. These were incubated for 1 day at 30°C, after which a Zeiss microscope (Axio Imager.M2) was used to take images of representative areas on the agar plate for further analysis (Supplementary Figure 2). Viability assays included three biological replicates per tested strain and were performed at two independent times (*n* = 6).

### Crystal Violet Assay

To assess fungal biofilm formation, i.e., surface-associated growth with production of extracellular matrix, a crystal violet assay was performed, adapted from Mowat et al. (2007). In a 96-well plate each well contained 100 μl spore suspension (to a total concentration of 10^5^ spores/ml per well), 100 μl MM, and 100 μl of distilled H_2_O (dH_2_O) (the controls contained an additional 100 μl MM instead of 100 μl spore suspension) and were incubated for 48 h at 30°C. After incubation, the wells were washed three times with dH_2_O, and 300 μl of crystal violet (0.5%) were added to each well to stain the surface-associated biomass (crystal violet stains hyphae and extracellular matrix that do not detach after washing). Excess staining was removed by washing with dH_2_O. De-staining was carried out by adding 300 μl of 95% ethanol. The absorbance of the ethanol-crystal violet solution was measured at 570 nm. The higher the absorbance value the greater the quantity of biological material. The absorbance was evaluated using the VICTOR Nivo Multimode Microplate Reader (PerkinElmer, Waltham, MA, United States). The assay included eight biological replicates per tested strain and was performed twice (*n* = 16).

### Data Analysis

Student’s *t*-test was performed to analyze the significance between individual data points where a two-tailed *p*-value ≤ 0.05, was considered significant. Error bars as standard error. Linear regression on survival fraction data was used to calculate the lethal dose for 90% of the population (LD_90_ values) – which is the same as D_10_ values (10% survivability).

## Results

### *Aspergillus niger* Spore Resistance Toward X-Ray Radiation

Spores from the wild-type, color mutant (Δ*fwnA)* and NHEJ mutant (Δ*kusA*) strains were exposed to different X-ray doses (up to 1000 Gy) in saline solution. The lethal dose required to inactivate 90% of the spores was similar for both wild-type (LD_90_ = 366 Gy) and Δ*fwnA* strains (LD_90_ = 353 Gy). After the maximum tested radiation dose of 1000 Gy, the Δ*fwnA* strain demonstrated no significant differences in survival when compared with the wild-type (5.3 × 10^3^ ± 1.3 × 10^3^ CFU/ml, versus 1 × 10^4^ ± 3.2 × 10^3^ CFU/ml). In contrast, at 500 Gy, Δ*kusA* was shown to be significantly more sensitive to X-ray radiation (1.25 × 10^4^ ± 1.1 × 10^3^ CFU/ml with a LD_90_ of 57 Gy), when compared to the wild-type (5 × 10^6^ ± 8.7 × 10^5^ CFU/ml) (*p* = 0.000). No CFU were detected above 500 Gy for Δ*kusA*. Survivability and LD_90_ values of the strains toward X-ray radiation, in all tested conditions, are shown summarized in Table 2.

**TABLE 2:** Lethal dose (LD_90_) values for *Aspergillus niger* spores irradiated with X-rays under different space-relevant conditions.

Strain	0.9% NaCl	H_2_O	Air-dried (0.9% NaCl)	Air-dried (H_2_O)
Wild-type	366 (*R*^2^ = 0.97)	362 (*R*^2^ = 0.96)	187 (*R*^2^ = 0.99)	204 (*R*^2^ = 0.99)
Color mutant (Δ*fwnA*)	353 (*R*^2^ = 0.98)	306 (*R*^2^ = 0.99)	175 (*R*^2^ = 0.99)	185 (*R*^2^ = 0.99)
NHEJ mutant (Δ*kusA*)	57 (*R*^2^ = 0.93)	55 (*R*^2^ = 0.92)	35 (*R*^2^ = 0.99)	45 (*R*^2^ = 0.95)

*Data reported as LD_90_ values – dose of radiation treatment leading to a 90% inactivation of the initial CFU (same as D_10_ values). Values in Gray (Gy).*

### Air-Drying of *A. niger* Spores Reduces Their Resistance Toward X-Ray Radiation

**FIGURE 1:**
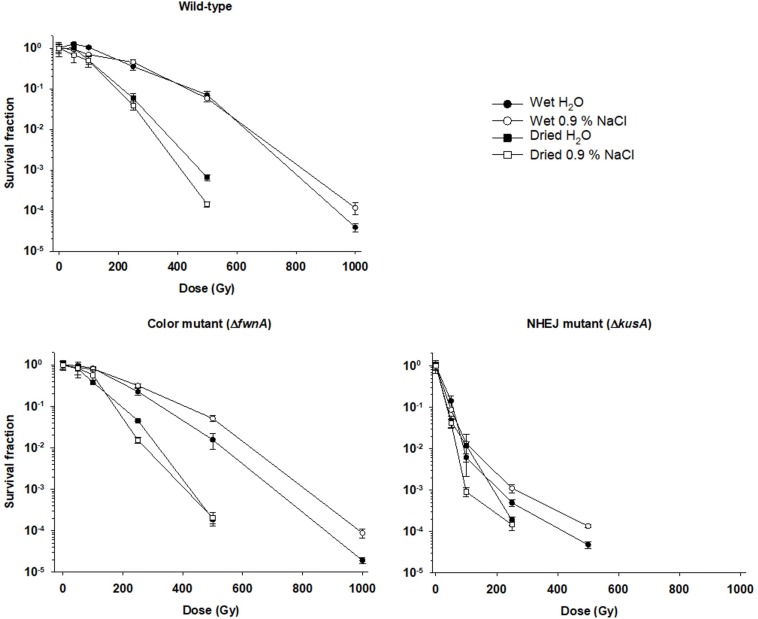
Effect of desiccation in survival to X-ray radiation of spores of different *Aspergillus niger* mutant strains. Spores were irradiated in either liquid suspensions (water or saline solution) or air-dried (from water or from saline solution). Survival fraction was calculated relatively to the non-irradiated controls.

Survivability of air-dried *A. niger* spores was compared with survivability of wet spores (both in water and saline solution). The highest X-ray dose at which there were detectable colonies was 500 Gy for both wild-type and Δ*fwnA* strains, and 250 Gy for Δ*kusA*. Results show that survivability of dried spores was decreased in comparison to wet spores (both in water and saline solution) in all tested strains (Figure 1). At 500 Gy, wild-type spores in water (4.9 × 10^6^ ± 1.1 × 10^6^ CFU/ml) survive better than dried spores from water (4.6 × 10^4^ ± 7.2 × 10^3^ CFU/ml) (*p* = 0.001). The same is seen for spores in saline solution where wet spores (5 × 10^6^ ± 8.7 × 10^5^ CFU/ml) survived better than dried spores from saline solution (8.2 × 10^3^ ± 1.2 × 10^3^ CFU/m) (*p* = 0.000) (Table 2). When comparing water- versus saline-dried spores, wild-type spores dried from saline solution (8.2 × 10^3^ ± 1.2 × 10^3^ CFU/ml) survive significantly less than spores dried from water (4.6 × 10^4^ ± 7.2 × 10^3^ CFU/ml) (*p* = 0.000). Loss of pigmentation did not affect resistance to desiccation (0 Gy) (*p* = 0.6); or radiation resistance (at 500 Gy) when spores were air-dried from saline solution (*p* = 0.4). However, loss of pigmentation decreased spore survival in irradiated spores dried in water: at 500 Gy, Δ*fwnA* spores were significantly more sensitive (5.2 × 10^3^ ± 1.2 × 10^3^ CFU/ml) than wild-type spores (4.6 × 10^4^ ± 7.2 × 10^3^ CFU/ml) (*p* = 0.002).

### *Aspergillus niger* Spores Resistance Toward Cosmic Radiation

**FIGURE 2:**
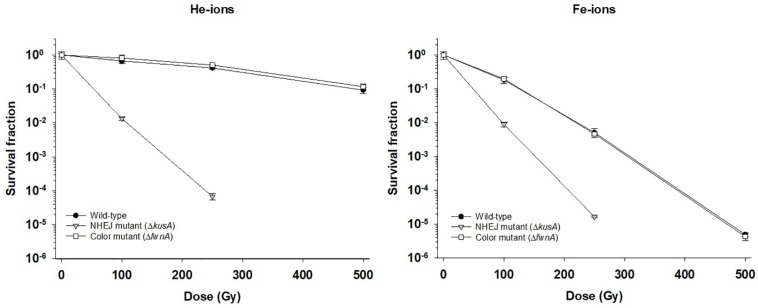
Effect of cosmic radiation (helium-ions – left; and iron-ions – right) on survival of spores of different *Aspergillus niger* strains. Survival fraction was calculated relatively to the non-irradiated controls.

**FIGURE 3:**
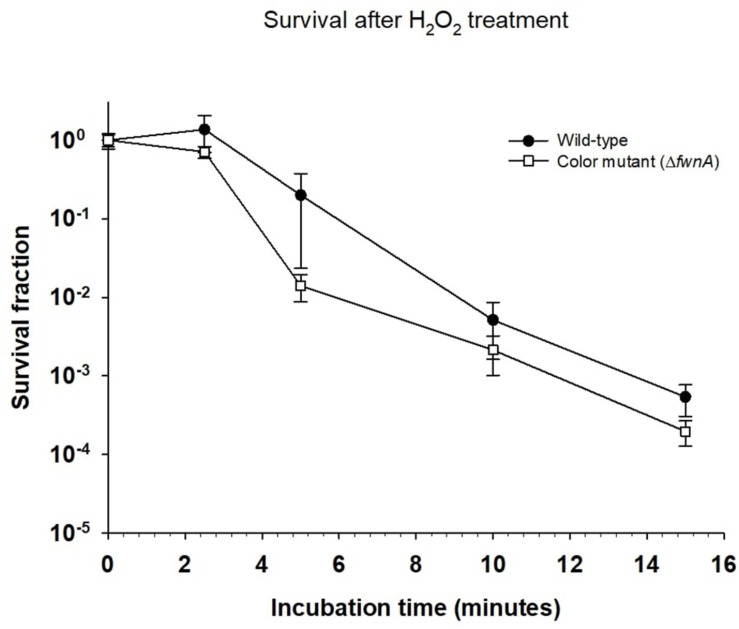
Effect of incubation with H_2_O_2_ on *Aspergillus niger* spore survival. Survival fraction was calculated relative to the non-treated (0 min) samples.

The effect of cosmic radiation on the survival of *A. niger* was tested by irradiating spores with helium- and iron-ions. Spores from the wild-type, color mutant (Δ*fwnA)* and NHEJ mutant (Δ*kusA*) strains were exposed to up to 500 Gy of helium- and iron-ions. At 500 Gy, wild-type spores survived less when irradiated with iron-ions (4.7 × 10^2^ ± 6.01 × 10^1^ CFU/ml) than when irradiated with X-rays (5 × 10^6^ ± 8.7 × 10^5^ CFU/ml) (*p* = 0.06), or helium-ions (8.7 × 10^6^ ± 1.9 × 10^6^ CFU/ml) (*p* = 0.01) (Table 3). The same trend holds for Δ*fwnA* spores at 500 Gy, where survival toward iron-ions (4.3 × 10^2^ ± 1.9 × 10^2^ CFU/ml) was decreased in comparison to both X-rays (3.4 × 10^6^ ± 5.4 × 10^5^ CFU/ml) (*p* = 0.004) and helium-ions (1.2 × 10^7^ ± 2.0 × 10^6^ CFU/ml) (*p* = 0.004) (Figures 1, 2 and Table 3). This is consistent with the fact that helium-ions are lighter elements in cosmic radiation whereas iron-ions are heavier particles, which cause greater damage to cells (Cucinotta and Durante, 2006). At 250 Gy, survival of Δ*kusA* spores was significantly reduced when irradiated with iron-ions (1.9 × 10^3^ ± 9.9 × 10^1^ CFU/ml) than when irradiated with X-rays (4.7 × 10^2^ ± 6.01 × 10^1^ CFU/ml) (*p* = 0.02), with no colony formation being observed above 250 Gy of cosmic radiation (Figure 2). Exposure to helium- and iron-ions (cosmic radiation) showed that both wild-type and Δ*kusA* spores were able to germinate after exposure to 250 and 500 Gy, respectively (Supplementary Figure 2), suggesting that there is a greater level of resistance if germination would be considered instead of colony forming ability. Since Δ*fwnA* spores do not show reduced resistance toward X-ray radiation (and subsequent ROS) in comparison to the wild-type strain, we tested whether pigmentation is involved in protecting the spore from H_2_O_2_-induced oxidative-stress by incubating the spores up to 15 min in 5% H_2_O_2_ (Figure 3). Both the wild-type and color mutant strains decreased in survival when incubated with H_2_O_2_. The survival of Δ*fwnA* spores after 15 min was lower than that of the wild-type, with the LD_90_ value for the wild-type strain (5 min) being higher than that of the Δ*fwnA* (3.1 min) (Figure 3).

**TABLE 3:** Lethal dose (LD_90_) values for *Aspergillus niger* spores irradiated with different types of ionizing radiation.

Strain	X-rays (Gy)	Helium-ion (Gy)	Iron-ion (Gy)
Wild-type	366 (*R*^2^ = 0. 97)	506 (*R*^2^ = 0.98)	112 (*R*^2^ = 0.99)
Color mutant (Δ*fwnA*)	353 (*R*^2^ = 0.98)	567 (*R*^2^ = 0.96)	112 (*R*^2^ = 0.99)
NHEJ mutant (Δ*kusA*)	57 (*R*^2^ = 0.93)	55 (*R*^2^ = 0.99)	50 (*R*^2^ = 0.99)

*Data reported as LD_90_ values – dose of radiation treatment leading to a 90% inactivation of the initial CFU (same as D_10_ values).*

### *Aspergillus niger* Spores Are Highly Resistant Toward UV-C Radiation

**FIGURE 4:**
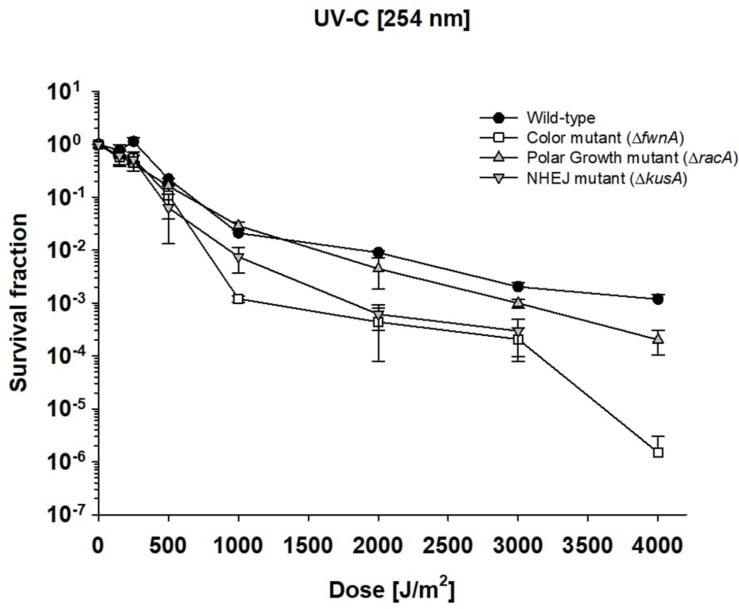
Effect of UV-C radiation in survival of *Aspergillus niger* spores. Survival fraction was calculated relative to the non-irradiated control samples. Error bars as standard error.

To investigate the impact of UV radiation on *A. niger* spore survivability, spore suspensions with 10^6^ spores/ml (spore monolayer) were exposed to 0–4000 J/m^2^ UV-C radiation (254 nm). At the highest tested dose of 4000 J/m^2^, both the wild-type (7.1 × 10^2^ ± 4.3 × 10^2^ CFU/ml) and polar growth mutant (4.2 × 10^1^ ± 2.5 × 10^1^ CFU/ml) demonstrated high survival (Figure 4). No CFU were detected for Δ*kusA* at 4000 J/m^2^. All tested strains were able to cope with 3000 J/m^2^ of UV-C exposure. At 3000 J/m^2^ wild-type spores (1.3 × 10^3^ ± 6.8 × 10^2^ CFU/ml) and Δ*racA* spores (2.3 × 10^2^ ± 1.3 × 10^2^ CFU/ml) displayed high survivability; whereas Δ*fwnA* spores (6.3 × 10^1^ ± 3.7 × 10^1^ CFU/ml) and Δ*kusA* spores (1.2 × 10^2^ ± 0.1 × 10^1^ CFU/ml) displayed low survivability. The UV-C dose required to eliminate 90% of the wild-type spores (LD_90_) was 1038 J/m^2^. LD_90_ of Δ*racA* spores was 826 J/m^2^, of Δ*kusA* spores was 580 J/m^2^, and for Δ*fwnA* spores it was 512 J/m^2^. The data also shows that deletion of *racA* increases survival toward UV-C (Figure 4), in all tested doses.

### Defect in Both NHEJ and Polar Growth Decreases *A. niger* Biofilm Formation

**FIGURE 5:**
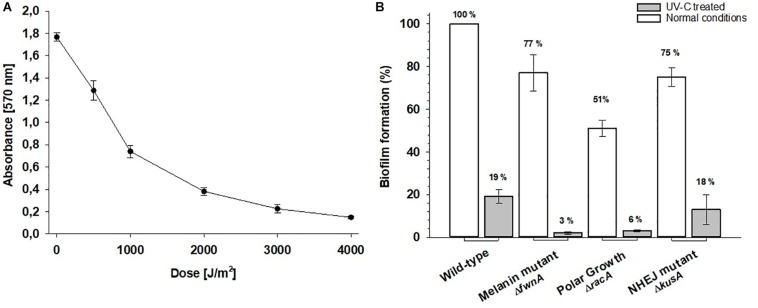
**(A)** Biofilm formation of *Aspergillus niger* wild-type after 0 to 4000 J/m^2^ UV-C (254 nm). Absorbance values are proportional to the amount of detected biomass. **(B)** Effect of UV-C treatment on *A. niger* biofilm formation. The biofilm formation is shown for normal conditions (white bars) and after treatment with 1000 J/m^2^ UV-C (gray bars).

When assessing *A. niger* biofilm formation (quantified as amount of surface-adhered biomass detected in the well after washing), a defect in pigmentation (Δ*fwnA*) decreased biofilm formation by 23% (*p* = 0.02); a defect in the NHEJ pathway (Δ*kusA*) decreased biofilm formation by 25% (*p* = 0.01); and a defect in both NHEJ and polar growth (Δ*kusA*, Δ*racA*) showed a decrease of 49% (*p* = 0.007) (Figure 5B). When assessing the effect of UV-C radiation in wild-type biofilm formation, we found that biomass decreased as doses increased up to 4000 J/m^2^ (Figure 5A). Thus, because the UV-C LD_90_ value for wild-type spores was 1000 J/m^2^, we tested biofilm formation after treatment with 1000 J/m^2^ UV-C for all strains. UV-C treatment led to 81% reduction in biofilm formation for wild-type spores (*p* = 0.04), 97% reduction in Δ*fnwA* spores, (*p* = 0.001), 82% in Δ*kusA* spores (*p* = 0.001), and 94% in Δ*racA*Δ*kusA* spores, (*p* = 0.005) (Figure 5B).

## Discussion

### Air Drying *A. niger* Spores Reduces Their Resistance Toward X-Ray Radiation

Spore survival toward X-rays was tested in both air-dried and liquid conditions (a comparison is provided in Table 4). Results show that air-dried spores have lower survival rates than spores irradiated in liquid suspension (either in water, or in saline solution) (Figure 1). This result is unexpected, as the presence of water is known to decrease radiation resistance via ROS formation. Nevertheless, *A. niger* spores have previously been shown to have increased radiation sensitivity when vacuum-dried and irradiated in air compared to wet spores irradiated in air (Silverman et al., 1967). However, the same study reported that vacuum-dried spores irradiated in vacuum were found to be more resistant to radiation, which implies that space vacuum desiccation might increase *A. niger* spore resilience (Silverman et al., 1967). Interestingly, a study assessing the impact of water in radiation resistance of yeast reports that small amounts of water substantially increases radiation sensitivity (Hutchinson et al., 1957). Thus, we consider the possibility that drying the spores overnight might have not been enough to retrieve all water from the spore suspension.

**TABLE 4:** Lethal dose (LD_90_) values for *Aspergillus niger* spores in comparison to different organisms in response to UV-C radiation (254 nm) and X-ray radiation. Values for cells/spores in suspension (wet), irradiated in air.

Strain	UV-C (J/m^2^)	References	X-rays (Gy)	References
*Aspergillus niger* (spores)	1038	This study	366	This study
*Bacillus subtilis* (spores)	100	Newcombe et al., 2005	857	Moeller et al., 2014
*Deinococcus radiodurans* (cells)	656	Bauermeister et al., 2009	ca. 8000	Moseley and Laser, 1965; Driedger et al., 1970

*Data reported as LD_90_ values – dose of radiation treatment leading to a 90% inactivation of the initial CFU (same as D_10_ values).*

Additionally, pigmentation did not have an effect on desiccation resistance (at 0 Gy), which is consistent with previous findings concerning the role of pigmentation in fungal survival to low water activity (Segers et al., 2018). When irradiating air-dried spores with 500 Gy, there was no difference between wild-type and color mutant for spores dried in saline. However, the color mutant had increased sensitivity when spores were dried in water. Thus, to fully understand the effect of desiccation (air- or vacuum-induced) on fungal spore resistance to extreme radiation further studies are required.

### High-Versus Low-LET Response in *A. niger* Spores

The biological effectiveness of radiation in a given biological sample is dependent on the LET. This means that equal doses of different types of radiation can induce different types of damage, and thus different cellular responses (Moeller et al., 2017). With this, previous studies with animals suggest that indirect damage is the main biological effect of low-LET radiations (such as X-rays or helium-ions), whereas direct damage is the main biological effect of high-LET radiation (such as iron-ions) (Kennedy, 2014). In contrast, the current study suggests that direct damage, in the form of DNA double-strand breaks, is induced by low-LET radiation exposure (X-rays and helium-ions), given that *A. niger* spore survivability was highly dependent on the NHEJ pathway (Δ*kusA*). Additionally, Δ*kusA* spores showed similar survival rates toward both high-LET iron-ion radiation (Figure 2) and low-LET X-ray radiation (Figure 1). This implies that radiation with iron-ions promotes additional cellular damages besides double-strand breaks, possibly as indirect damage in the form of ROS generation. Multiple experiments have shown similar results in yeasts exposed to ion radiation (Ikpeme et al., 1995; Kiefer et al., 2002). Conversely, a study with the filamentous fungus *Neurospora crassa*, showed that an NHEJ-deficient strain had ∼80% survival after 100 Gy of high-LET carbon-ion irradiation (Ma et al., 2013), whereas the NHEJ-deficient *A. niger* strain tested in the current study demonstrated survival of only ∼17% after 100 Gy of low-LET helium-ion radiation (a lighter element than carbon). Nevertheless, this discrepancy in survival might be attributed to the fact that hyphal compartments of *N. crassa* can contain up to 100 nuclei, which in turn can lower the hit rate of radiation induced DNA damage (Roper et al., 2011).

### High Resistance of *A. niger* Spores Toward UV-C

UV-derived decontamination methods are commonly used in modern laboratories and healthcare systems (Yang et al., 2019), with UV-C lamps used to sterilize biosafety cabinets reaching around 300 J/m^2^ in 12.5 min (Meechan and Wilson, 2006). Yet, in this study *A. niger* wild-type spores demonstrated high resistance toward UV-C (Figure 4, and Supplementary Figure 3), where the lethal dose required to eliminate 90% of wild-type spores was 1038 J/m^2^. This is significantly higher than the LD_90_ of other microorganisms (Table 4), and becomes particularly clear in comparison with previously characterized radiation-resistant organisms such as *Deinococcus radiodurans* (LD_90_ = 660 J/m^2^) or *B. subtilis* spores (LD_90_ = 100 J/m^2^). In a study using UV-C to treat drinking water, *A. niger* was found to be completely inactivated after exposure to 1920 J/m^2^ UV-C (Sisti et al., 2017), which is in agreement with our study (LD_90_ = 1038 J/m^2^).

The NHEJ pathway was shown to be an important DNA repair mechanism for survival of *A. niger* spores after exposure to UV-C and X-ray radiation. Interestingly, previous studies with NHEJ mutants of *N. crassa* and *Cryptococcus neoformans* did not show differences in survival after UV-C irradiation when compared to the wild-type (Ninomiya et al., 2004; Goins et al., 2006). It is possible that point mutations and single-strand breaks caused by UV radiation can be repaired by KusA independent repair mechanisms (Eckardt-Schupp and Klaus, 1999; Goldman et al., 2002). However, the higher nucleus number per hyphal compartment in *N. crassa*, and the capsule formation of *C. neoformans* spores (McFadden and Casadevall, 2001) may contribute to their increased radiation tolerance despite mutations in this repair pathway.

### Pigmentation as Key-Protection Against UV-C but Not X-Rays or Cosmic Radiation

Pigments, such as melanin are involved in different cellular processes from adhesion to virulence (Eisenman and Casadevall, 2012), and are known to help cells against radiation-induced stress and ROS (Cockell and Knowland, 1999). Studies concerning the role of pigmentation in radiation resistance of fungal spores have been performed with monochromatic UV-C (254 nm) and pulsed light up to 1770 J/m^2^ on inoculated agar with concentrations up to 10^7^–10^8^ spores/ml (Esbelin et al., 2013). The current study irradiated spore suspensions in liquid, using a concentration of 10^6^ spores/ml. This concentration was chosen to guarantee a spore monolayer and prevent self-shielding (Figure 4). As expected, pigment-deficient spores (Δ*fwnA*) demonstrated lower survivability after exposure to UV-C radiation. Interestingly, deficiency in pigmentation did not alter survivability after exposure to ionizing radiation (X-rays or cosmic radiation) (Figures 1, 2). This seems to contradict previous studies where melanin was shown to have a protective effect against X-ray irradiation in the fungi *C. neoformans* and *Cryomyces antarcticus* (Pacelli et al., 2017). However, it is to note that the strain tested in the current study lacks a putative polyketide synthase which results in fawn-colored (not white) spores, which might provide sufficient amount of pigmentation to display protective properties.

Both ionizing radiation and hydrogen peroxide are known to affect cell survival through the generation of ROS. To better understand how pigment-deficient spores resist to ionizing radiation, these were incubated in hydrogen peroxide. Results show that Δ*fwnA* spores were more sensitive to hydrogen peroxide than wild-type spores (Figure 3). This indicates that pigmentation is involved in protecting the spore from H_2_O_2_-induced oxidative-stress, but not in X-ray- or cosmic radiation-induced oxidative stress. Previous studies analyzing the effect of H_2_O_2_ on *A. niger* spores were able to show that ROS scavenging was facilitated by increased catalase expression resistance (Angelova et al., 2005). From the results presented here, contrary to what has been suggested, pigmentation does not influence *A. niger* survival to space-like ionizing radiation.

### UV-C Radiation Decreases Biofilm Formation Effectively

To address the contamination risks and possible decontamination procedures concerning *A. niger* growth in the spaceflight environment, wild-type spores were exposed to up to 4000 J/m^2^ of UV-C and we assessed the impact of UV-C on surface-associated growth, i.e., biofilm formation (Figure 5A). Because wild-type spores were 90% inactivated at a dose of 1000 J/m^2^, the ability for biofilm formation was assessed, for all strains, before and after treatment with 1000 J/m^2^ UV-C. Here, one additional strain, deficient in polar growth, was included. This strain lacks the Rho GTPase RacA involved in establishing polarized tip extension via regulation of the actin filaments, which is important for proper cell wall formation in *A. niger* hyphae (Kwon et al., 2011, 2013). Results show that Δ*racA* spores ability for biofilm formation was reduced by 49% before UV-C treatment, and 94% after UV-C treatment, when compared with the wild-type (Figure 5B). The underlying molecular mechanism for reduced surface-associated growth (before UV-C) might be due reduced adhesion to hydrophobic surfaces, and/or results in less spore aggregation during spore outgrowth – a hypothesis worth studying further. The reduction of surface-associated growth after exposure to radiation suggests that the function of RacA plays a role (direct or indirect) in UV-C resistance of *A. niger*. Moreover, the tested color mutant (Δ*fwnA*) strain also demonstrated decreased ability of surface-associated growth, both before and after UV-C treatment, which suggests the involvement of pigments in spore adhesion.

## Conclusion

This study shows that spores of *A. niger* are extremely resistant to space radiation. Spores were able to withstand high doses of X-ray (LD_90_ = 360 Gy), cosmic radiation (helium-ion LD_90_ = 500 Gy; and iron-ion LD_90_ = 100 Gy), and UV-C radiation (LD_90_ = 1038 J/m^2^). *A. niger* spore resistance to UV-C is particularly interesting, given that it is even higher than that of other radiation-resistant microorganisms (e.g., *D. radiodurans*). Air-drying the spores made them more susceptible to X-ray radiation. Moreover, wild-type spores treated with 1000 J/m^2^ UV-C were shown to have decreased biofilm formation ability (81% reduction). It is important to note that the ionizing radiation doses used in this study (up to 1000 Gy) are multiple times higher than doses expected from traveling in interplanetary space. For example, a 360-day round-trip to Mars would yield a dose of 0.66 ± 0.12 Gy (Zeitlin et al., 2013). It is therefore unlikely that *A. niger* spores become easily inactivated due to space radiation alone. We thus recommend that current planetary protection guidelines are revisited to address the high resistance of fungal spores in space travel scenarios. In addition, further studies are needed in order to address fungal spore resistance to other space environmental factors such as vacuum, changes in pressure, and extreme temperature fluctuations.

## Data Availability Statement

All datasets generated for this study are included in the article/Supplementary Material.

## Author Contributions

MC, AH, and RU performed the experiments, analyzed the data, and wrote the manuscript. VM, TS, RM, and AF contributed to the conception and design of the study, and manuscript preparation.

## Conflict of Interest

The authors declare that the research was conducted in the absence of any commercial or financial relationships that could be construed as a potential conflict of interest.
