# Supplementary material for: Aspergillus niger Spores Are Highly Resistant to Space Radiation
Source: Front Microbiol. 2020 Apr 3;11:560. doi: 10.3389/fmicb.2020.00560 (PMC7146846; doi:10.3389/fmicb.2020.00560)
Supplement: Supplementary file 1 [file Data_Sheet_1.PDF]

Supplementary Material

1 Supplementary Figures

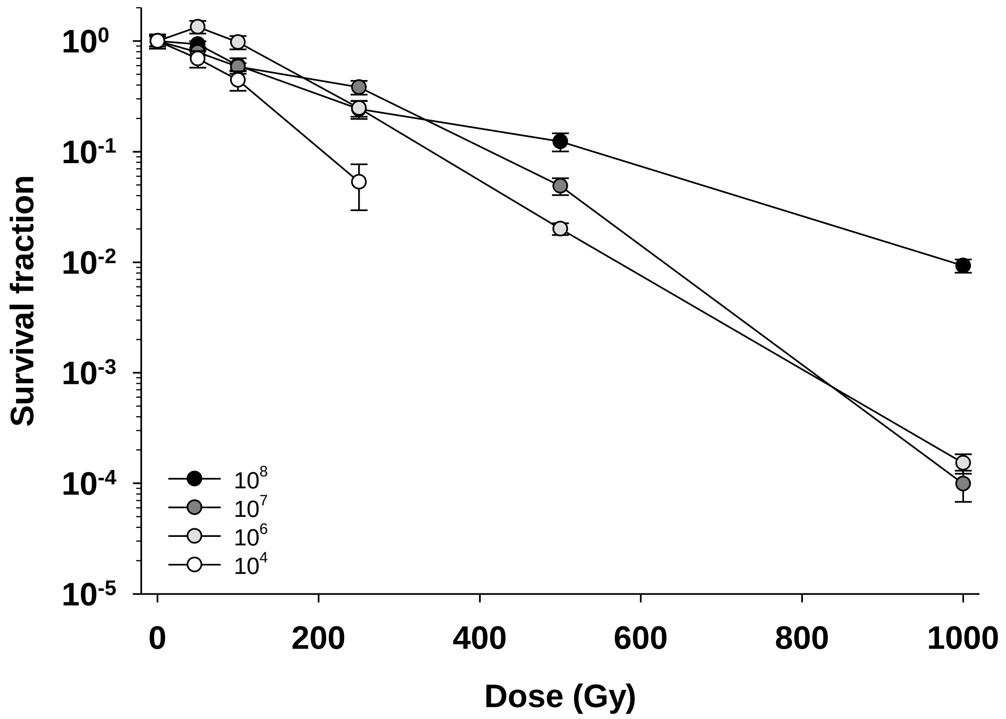

Supplementary Figure 1. Effect of initial spore concentration (inoculum) in survivability towards ionizing (X-ray) radiation. At 10^8 spores/ml there is a spore multi-layer that can provide radiation shielding, whereas at 10^6 and 10^7 spores/ml there is a spore monolayer, preventing self-shielding from the spores. No CFU were detected after 250 Gy for 10^4 spores/ml.

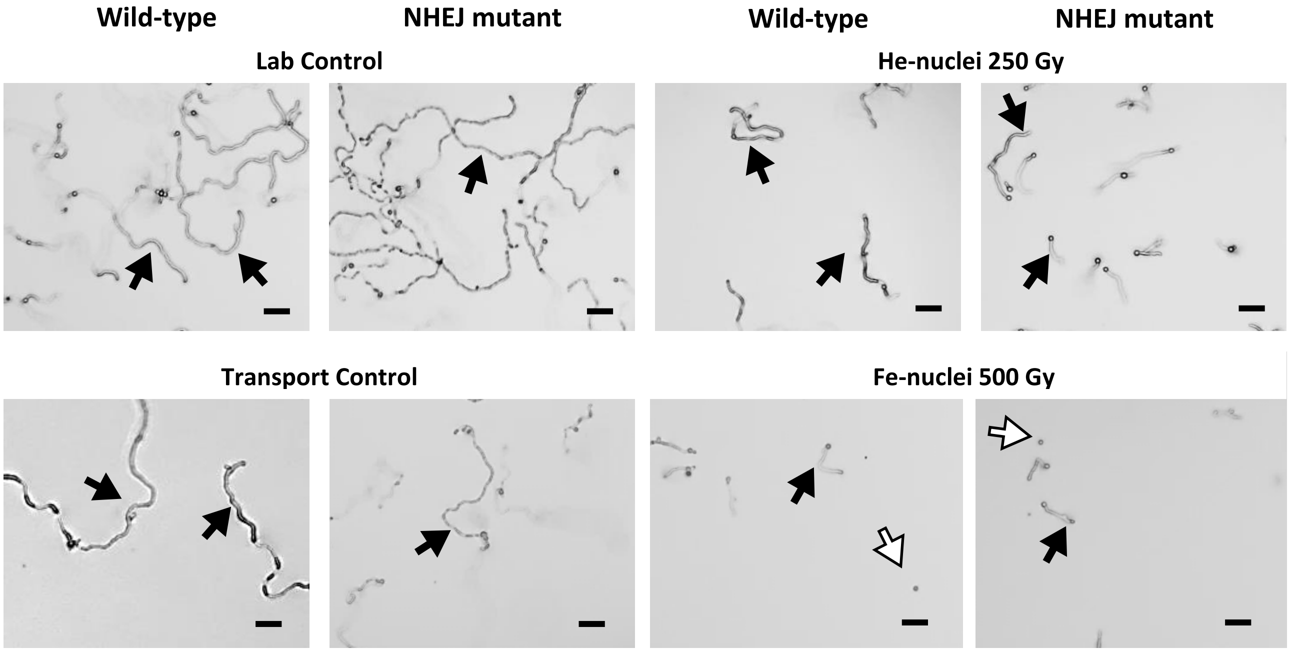

Supplementary Figure 2. Germination of *A. niger* spores after He- and Fe-nuclei irradiation. Black arrows indicate regions of interest showing spore germination and varying hyphal length as the radiation dose increases. At 500 Gy of Fe-nuclei radiation there CFU were detected, but spore germination can still be identified. White arrows indicate un-germinated spores. Pictures were taken with after one day of incubation on MM-agar plates with an Axio Imager. M2 at 100 x magnification. Each picture was chosen as representative for the respective condition. Scale bar: 50 µm.

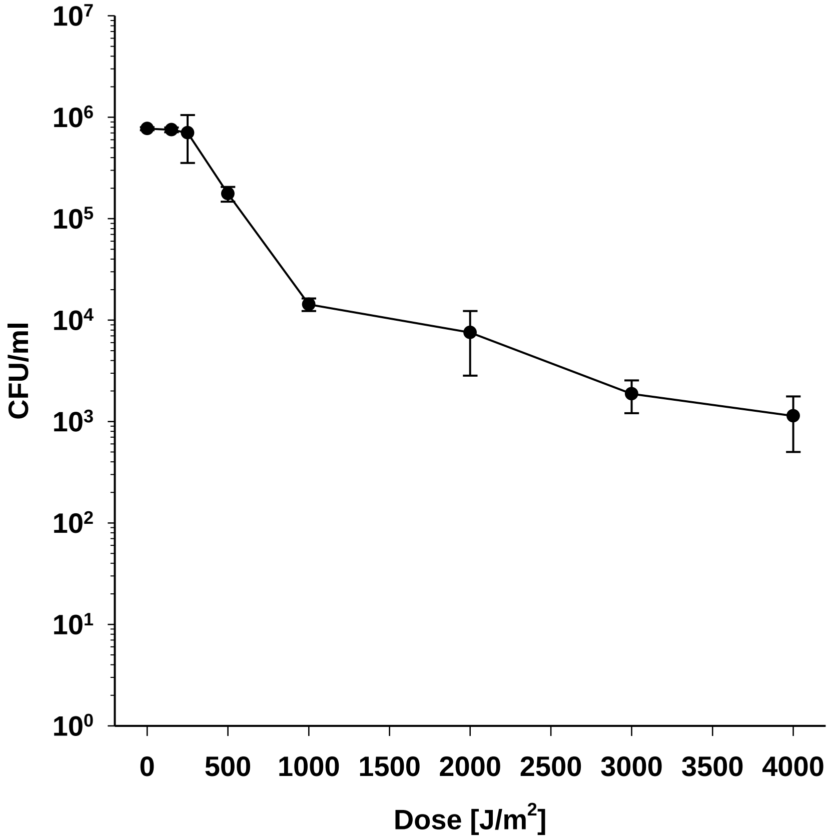

Supplementary Figure 3. UV-C survival curve of *Aspergillus niger* wild-type spores showing dose-effect. Spores irradiated in 0.9 % NaCl (10^6 spores/ml), in air. Survivability was calculated as colony-forming units (CFU) per ml. LD_{90} value is 1038 J/^{m2}.
